# Stability breakthrough accelerates commercialization of perovskite indoor photovoltaics

**DOI:** 10.1093/nsr/nwaf409

**Published:** 2025-10-13

**Authors:** Weiwei Zuo, Michael Saliba

**Affiliations:** Institute for Photovoltaics, University of Stuttgart, Germany; Institute for Photovoltaics, University of Stuttgart, Germany; Helmholtz Young Investigator Group FRONTRUNNER, IMD3-Photovoltaik, Forschungszentrum Jülich, Germany

The rapid expansion of the Internet of Things (IoT) has driven the widespread deployment of wireless sensors, e.g. in indoor environments. The growing demand for large data sets to train AI systems underscores the need for low-power, self-sustaining devices [1]. Conventional battery-powered solutions have limited lifespans and raise environmental concerns, underscoring the importance of autonomous energy harvesting.

However, indoor lighting conditions differ significantly from natural sunlight in terms of spectrum and intensity imposing unique demands on indoor photovoltaics [2]. For example, indoor spectra typically do not come with large portions of red photons, thus shifting the ideal bandgap of the active material required for optimal power output towards the blue.

Perovskite indoor photovoltaics (PIPVs) in particular offer advantages such as tunable band gaps and high-voltage outputs [3], making them ideal for indoor energy harvesting. Self-assembled monolayers (SAMs) are often used to adjust the perovskite lattice and improve photothermal stability, which is essential for extending the lifespan of devices [4]. Nevertheless, their susceptibility to degradation under thermal stress and light-dark cycling introduces a new reliability bottleneck [5]. Therefore, developing thermally and optically stable SAM layers is crucial for improving PIPV performance and ensuring long-term operational stability in IoT applications.

**Figure 1.**
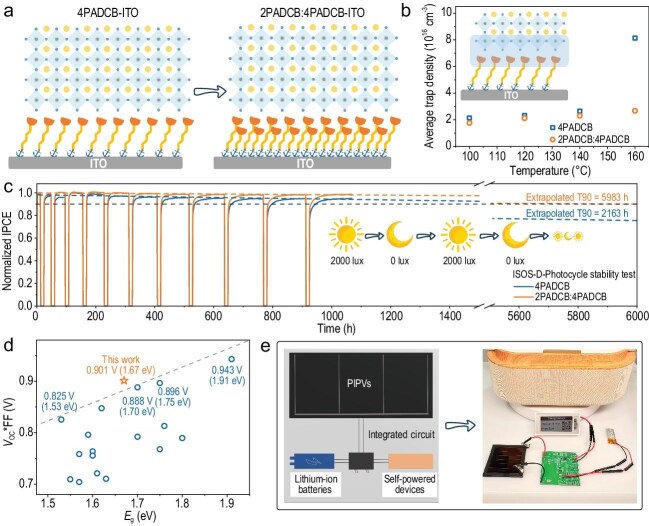
(a) Schematic diagram of 4PADCB and interlocking SAM morphology. (b) Average trap density of the buried interface of perovskite films. (c) ISOS-D-Photocycle stability test of the pristine and target PIPVs. (d) Summary of *V*_OC_*FF of the published PIPVs in recent years. (e) The integrated circuit diagram, including integrated circuit boards, PIPVs, lithium batteries and self-powered devices such as electronic price tags. The right photo shows electronic price tags driven by the integrated circuit diagram. Adapted from Ref. [4] with permission.

In a recent study, Zhao-Kui Wang’s group introduced an interlocking SAM strategy blending short-chain (2PADCB) and long-chain (4PADCB) carbazole-phosphonic acid molecules at a 1:1 ratio [6]. As illustrated in Fig. 1a, the shorter 2PADCB molecules fill voids within the 4PADCB matrix, boosting ITO surface coverage by 17% and strengthening interfacial adhesion. The mechanism underlying this stability leap was revealed through trap density analysis: after 160°C

thermal stress, interfacial trap density in interlocked-SAM devices rose only 52% compared to a 280% surge in controls (Fig. 1b), demonstrating superior resilience against degradation.

Crucially, the interlocking SAM design dramatically extended device longevity. Accelerated aging tests simulating day/night illumination cycles (2000–0 lux) projected a T90 operational lifetime of 5983 hours—nearly triple that of conventional SAM-based devices (2163 hours, Fig. 1c). This breakthrough in molecular engineering also yielded exceptional performance: optimized devices achieved a record indoor power conversion efficiency (IPCE) of 42.01% under 1000-lux LED illumination, with a high open-circuit voltage of 1.07 V and fill factor of 84.22%—surpassing other reported values for devices with similar perovskite bandgaps (Fig. 1d). The elevated voltage parameters critically facilitate stable operation of self-powered IoT devices by meeting their minimum drive requirements.

Validating real-world applicability, the researchers integrated PIPV modules with lithium-ion batteries and control circuits to power self-sustaining electronic price tags (Fig. 1e). This practical demonstration, coupled with the efficiency-stability synergy, positions PIPVs as a transformative energy solution for the IoT ecosystem.
